# LiveSeq: A New Technique to Sample RNA From Cells Without Killing Them

**DOI:** 10.1097/HS9.0000000000000834

**Published:** 2023-02-17

**Authors:** David G. Kent

**Affiliations:** 1 York Biomedical Research Institute, Department of Biology, University of York, United Kingdom

One of the most fundamental challenges facing stem cell biologists over the decades has been the need to destroy a cell to prove that it is actually a stem cell. This makes assays that require destroying cells impossible to run in tandem with a functional stem cell assay, thereby preventing researchers from studying the stem cell state. This summer, a very exciting paper was published that purports to break through this decades-old challenge. The technique is called “LiveSeq” and it enables researchers to extract a tiny amount of mRNA from cells without compromising cell function or survival.

Previous efforts in this space have either relied on low-parameter cell marking (eg, flow cytometry, reporter genes), split progeny of single cells and sampled separately,^1,2^ or attempted to use index-sorting to link cells computationally in a retrospective fashion.^3,4^ None of these were able to undertake global gene expression analyses in the same cell that could then continue to demonstrate its functional capacity after the sampling period. The ability to sample live cells would be a massive breakthrough for such studies and LiveSeq gives us the first glimpse into this becoming a reality for biologists.

As with all things, if it were easy to do then someone would have already done it and LiveSeq is certainly not easy by any definition. It is based on a technology known as fluidic force microscopy which is effectively an atomic force microscope with the ability to dispense picolitre volumes of liquid. This also allows it to act as a very tiny syringe whereby you can extract tiny amounts of liquid from a cell. This is where the magic comes in—that extraction does not have to kill the cell. In their landmark study entitled “Live-seq enables temporal transcriptomic recording of single cells,” Chen et al^5^ not only showed that cells could survive the sampling but also undertook experiments to demonstrate that cell function was not altered by the sampling. Specifically, they assayed individual macrophages exposed to lipopolysaccharide (LPS) and individual adipose stromal cells pre- and post-differentiation. In the case of primary mouse adipocytes, 44 individual cells were sampled longitudinally across 7 days and a few important findings were described: (1) there was no significant drop in survival of cells that had undergone extraction (95%) versus those that had not (93%) and differentiation (as measured by lipid droplet formation) was maintained to similar levels, suggesting that sampling did not impact survival or differentiation. In the case of the macrophages, substantial heterogeneity in LPS response was observed and the researchers were able to analyze the individual transcriptomes as a function of response. This revealed a previously unknown role of *Nfkbia* in LPS response and underscores the power of being able to sample RNA and perform a functional assay in the same cell.

Despite this amazing capability, there are some definite kinks to iron out. First, the depth of the transcriptome was lower across all cell types sampled and it appeared that roughly 60% of the genes were captured in LiveSeq compared with smart seq II. Second, assays selected for validation in new cell types of interest may not be as favorable after LiveSeq sampling. Third, it takes a bit of time to perform—the methods state an average of 15 minutes to load buffer, select a cell, extract the biopsy and transfer to lysis buffer (with an average of 10–20 biopsies per experiment). Fourth, the equipment and expertise required and experimental setup is not trivial as it requires an atomic force microscope and the protocol has many carefully worked out steps. These are not insubstantial hurdles in and of themselves and each one will likely take teams of researchers to optimize going forward.

All that said though, this cracks open a huge range of new experiments where a single cell can be sampled and then coupled to the functional output of the cell, building a link that has been out of reach for scientists up until this point. This nondestructive global gene expression tool for sampling the transcriptome of live cells will almost certainly be utilized in additional settings and the next few years will be an exciting time to see how far this technology can be pushed (and democratized!).

## AUTHOR CONTRIBUTIONS

DGK conceptualized, wrote, and edited the manuscript.

## DISCLOSURES

The author has no conflicts of interest to disclose.
